# Diagnostic accuracy and risk stratification of the score for trauma triage in the geriatric and middle-aged among older adults with fall-related injuries

**DOI:** 10.1371/journal.pone.0338948

**Published:** 2025-12-18

**Authors:** Oluwaseun John Adeyemi, Sanjit Konda, Charles DiMaggio, Corita R. Grudzen, Ashley Pfaff, Garrett Esper, Mauricio Arcila-Mesa, Allison M. Cuthel, JohnRoss Rizzo, Jean-Baptiste Bouillon-Minois, Helen Poracky, Polina Meyman, Ian Wittman, Joshua Chodosh

**Affiliations:** 1 Ronald O. Perelman Department of Emergency Medicine, NYU Grossman School of Medicine, New York, United States of America; 2 Department of Orthopedic Surgery, NYU Grossman School of Medicine, New York, United States of America; 3 Department of Surgery, NYU Grossman School of Medicine, New York, United States of America; 4 Department of Population Health, NYU Grossman School of Medicine, New York, United States of America; 5 Department of Medicine, Memorial Sloan Kettering Cancer Center, United States of America; 6 Department of Rehabilitation Medicine, NYU Grossman School of Medicine, New York, United States of America; 7 Department of Emergency Medicine, University Hospital of Clermont-Ferrand, CHU, Clermont-Ferrand, France; 8 Department of Trauma, NYU Langone Hospital, New York, United States of America; 9 Department of Medicine, NYU Grossman School of Medicine, New York, United States of America; 10 Medicine Service, Veterans Affairs New York Harbor Healthcare System, New York, New York, United States of America; Keio University School of Medicine, JAPAN

## Abstract

**Background:**

Despite fall-related injuries accounting for over two-thirds of older adult trauma injuries, fall-related injuries are more likely to be under-triaged. The Score for Trauma Triage in the Geriatric and Middle-Aged (STTGMA) is an injury risk-triage tool. This study aims to validate STTGMA’s accuracy in predicting fall-related mortality among older adult trauma patients and compare its predictive accuracy with the Geriatric Trauma Outcome Score (GTOS) and the Revised Trauma Score (RTS).

**Methods:**

Using a retrospective cohort design, we selected 6,458 older adult trauma patients (aged 65 years and older) from a single institutional trauma database (2017–2023). The primary outcome variable was in-hospital death, measured as a binary variable. The primary predictor variable was the STTGMA score, measured as a continuous variable and a four-level categorical variable. The secondary predictor variables were the GTOS and the RTS. We compared the predictive accuracy (95% confidence interval (CI)) of the STTGMA, GTOS, and RTS. We further assessed the relationships between the STTGMA risk categories and time-to-death and hospital length of stay using multivariable time-varying Cox proportional hazard analysis and multivariable quantile regression analysis, respectively.

**Results:**

A total of 130 patients (2.0%) died during admission, and the median hospital length of stay was 2 days. STTGMA exhibited 84% (95% CI: 77.3–89.8) accuracy in predicting in-hospital fall-related mortality, while the GTOS and RTS both exhibited 71% diagnostic accuracies. Compared to the minimal risk category, older adult trauma patients classified as low, moderate, and high risks each had significantly longer hospital stays and adjusted mortality risks, in a dose-response pattern.

**Conclusion:**

STTGMA can accurately predict in-hospital mortality and risk-stratify the length of stay and the time to death among older adult trauma patients with fall-related injuries.

## Introduction

Falls are the leading cause of injury-related mortality among U.S. older adults (aged 65 years and older), and every day, approximately 90 older adults die from fall-related injuries [1]. Between 2007 and 2016, the age-adjusted fatal fall rate among older adults increased by approximately 30 percent, from 47 to 62 deaths per 100,000 population, with rates highest among adults 85 years and older [2,3]. With 10,000 US adults turning 65 years daily [4], fall-related injuries are expected to increase. Currently, fall-related injuries account for approximately 800,000 emergency department (ED) visits annually, and the U.S. spends over $750 million yearly on fatal fall-related injuries [3].

Despite fall-related injuries accounting for two-thirds of older adult trauma injuries [5], fall-related injuries are more likely to be under-triaged [6]. The underestimation of fall-related injury severity among older adults further predisposes these patients to increased morbidity and mortality [6–8]. Pre-hospital injury triage tools such as the Ohio geriatric guideline and the Manchester triage guideline have demonstrated 68% and 74% accuracy in predicting geriatric trauma mortality, respectively [9,10]. In the non-US older adult trauma population, the diagnostic accuracies of the Geriatric Trauma Outcome Score (GTOS) [11], and Revised Trauma Score (RTS) [12,13], vary widely from 66 to 88% [14,15], and 82–95% [16,17], respectively. This variability in the diagnostic accuracies may be attributed to the diverse injury mechanisms within each study population, with a notable proportion experiencing motor vehicle crash injuries as opposed to falls [14–16,18]. Among the U.S. older adult trauma population, few studies have reported the diagnostic accuracies of the GTOS and RTS in predicting mortality [19,20], and none of these studies focused solely on fall injury-related mortality. With falls accounting for 75 percent of U.S. geriatric trauma injuries [21,22] and are 50 percent less likely to be associated with mortality compared to crash injuries [23], the knowledge of the diagnostic accuracy of these risk triage tools in predicting fall-related mortality is important for clinical practice and academic research.

The Score for Trauma Triage in Geriatric and Middle Age (STTGMA) is a risk triage tool that predicts mortality risk using age, injury severity characteristics, and background comorbidities [24]. The STTGMA risk triage tool has primarily been used in the older adult orthopedic trauma population to predict mortality from fracture-related injuries [25,26]. It has also been implemented to predict in-hospital disposition, the need for a blood transfusion, and the cost of care among orthopedic trauma patients [27–31]. Across these studies, the STTGMA risk triage tool has demonstrated 74–94% accuracy in predicting in-hospital mortality among patients with fracture-related injuries [25,26].

Assessing STTGMA’s predictive accuracy for fall-related mortality may validate the usability of the instrument for the care of older adult trauma patients. No study has assessed the sensitivity, specificity, and diagnostic accuracy of the STTGMA risk-triage tool among U.S. older adult trauma patients with fall-related injuries. Additionally, comparing the STTGMA risk triage tool to other common risk triage tools, such as the GTOS and RTS, will inform the predictive accuracy of these tools for fall-related mortality. Furthermore, investigating the relationship between STTGMA and other clinical measures of fall-related mortality, such as hospital length of stay and time-to-death, may further enhance its in-hospital utility. This study, therefore, has two aims. First, we sought to compare the diagnostic accuracies of the STTGMA risk triage tool, the GTOS, and RTS in predicting in-hospital fall-related mortality. Our null hypothesis was that there would be no statistically significant difference in the diagnostic accuracy between the STTGMA and the two other risk triage scores – GTOS and RTS. Secondly, we assessed the association between the STTGMA-derived risk categories and hospital length of stay and time-to-death from fall-related injuries.

## Methods

### Study design and setting

Using a retrospective cohort design, we pooled trauma registry data between January 2017 and June 2023 from a single Level I trauma center that serves a racially diverse community in New York. Data were extracted in August 2023, initially analyzed in September 2023, and subsequent analyses were performed as needed. This study is among the validation studies focused on exploring the diagnostic accuracy of a novel scoring tool for older adult trauma patients. The results we present follow the reporting guidelines of the Standards for Reporting Diagnostic Accuracy [32].

### Inclusion and exclusion criteria

**Fig 1:**
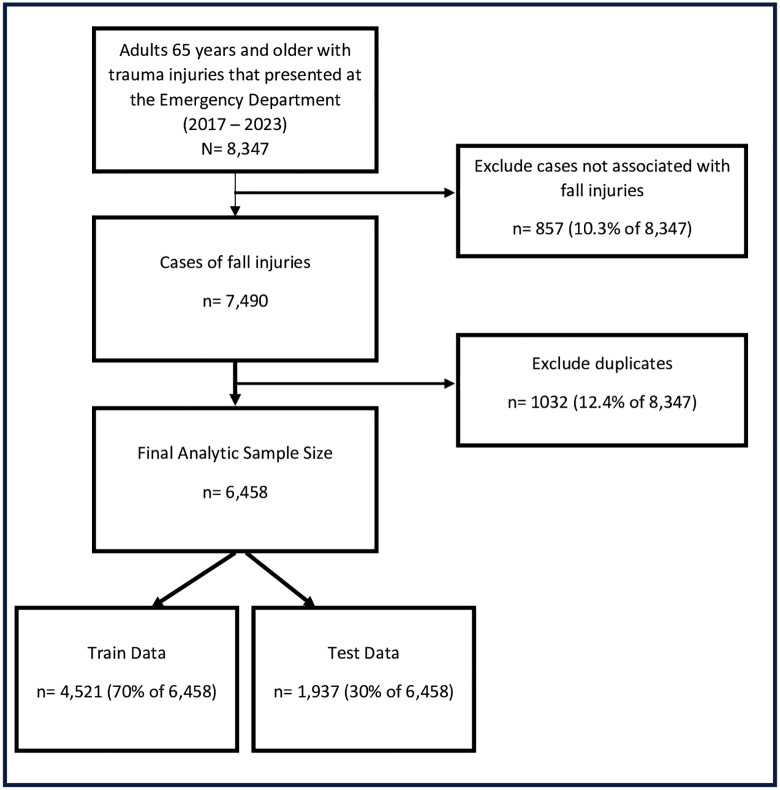
Data selection steps.

We selected adults, 65 years and older, who presented to the ED with traumatic injuries (N = 8,347) (Fig 1). We excluded patients whose injuries were not related to falls (n = 857; 10.3% of 8,347). We identified duplicate entries representing patients with multiple ED visits due to recurrent fall-related injuries. We selected the most recent fall, coded these patients as having recurrent ED visits due to falls, and excluded multiple entries (n = 1,032; 12.4% of 8,347). The final analysis included 6,458 adults aged 65 years and older who presented to the ED with fall-related injuries.

### Handling of missing data

Six variables had missing values – race/ethnicity (n = 345, 5.3%), body mass index (n = 646, 10.0%), Glasgow Coma Scale score (n = 548, 8.5%), systolic blood pressure (n = 328, 5.1%), respiratory rate (n = 326, 5.0%), and length of stay (n = 73, 1.1%). We performed multiple imputations after confirming that the missingness pattern was missing at random using Little’s test [33–35]. The multiple imputations used the multivariate normal regression with 100 iterations, and the imputed value represents the average across the 100 iterations [36,37].

### Data

From the analytic dataset (n = 6,458), we created two separate pools of data: the training dataset (n = 4,521) and the test dataset (n = 1,937). The training dataset was used for the model development and prediction analysis, while the test dataset was used for the Receiver Operating Characteristic (ROC) analysis. The split was done at a 70:30 ratio, using simple random sampling without replacement.

### Outcome variables

The primary outcome measure of interest was in-hospital death. We defined in-hospital death as death occurring either in the ED or during the index admission. In-hospital death was measured as a binary variable (yes/no), and this variable served as the outcome of interest for model prediction and ROC analyses. The secondary outcome measures of interest were hospital length of stay and the time to death from fall-related injuries. The hospital length of stay was defined as the duration from hospital admission to final hospital disposition. The final hospital disposition was defined as either discharge to home or facility, transfer to another hospital or facility, or death. Hospital length of stay was measured as a continuous variable. Time to death from fall-related injury was defined as the time from admission following the injury to the time of in-hospital death. Patients who were alive at the time of discharge were considered to have right-censored events.

### Predictor variables

The primary predictor variables were the STTGMA risk-triage score and the STTGMA risk categories. The STTGMA risk-triage score was computed using an online Excel calculator [24]. First, we determined if the injury was a high or low-energy mechanism. High-energy falls were falls from heights greater than two meters, while low-energy falls were falls from a standing height or lower. Thereafter, we calculated the STTGMA score using the patient’s age, Glasgow Coma Scale (GCS) score, Charlson Comorbidity Index (CCI), and Abbreviated Injury Scale (AIS) scores for the head and neck, chest, extremities, and pelvis. Age, AIS, GCS, injury severity score (ISS), chronic comorbid conditions, blood pressure, respiratory rate, and blood transfusion were obtained as pre-coded fields from the institutional trauma registry, which is maintained in accordance with the National Trauma Data Bank (NTDB) data definitions and coding standards. The trauma registry data were entered by certified registrars following standardized protocols and underwent routine quality audits prior to our data request. We computed the CCI (excluding age) as follows: 1 point each for myocardial infarction, congestive heart failure, peripheral vascular disease, cerebrovascular disease, dementia, chronic pulmonary disease, connective tissue disease, peptic ulcer disease, mild liver disease, and diabetes (2 points if with end organ damage); 2 points each for hemiplegia, moderate or severe renal disease, any tumor without metastasis (6 points if metastatic), leukemia, and lymphoma; 3 points for moderate or severe liver disease; and 6 points for Acquired Immunodeficiency Syndrome (AIDS) [38,39].

Each study participant had a single final STTGMA score, depending on whether the injury was high- or low-energy. The high-energy STTGMA risk-triage score was calculated using the formula: STTGMA_HE _= 1/ (1 + EXP(-(−8.6880 + 0.1133*Age – 0.3667*GCS + 0.5679*AIS_Head_and_Neck + 0.4146*AIS_Chest + 0.4629*AIS Extremities_and_Pelvic Girdle))). The low energy STTGMA risk-triage score was calculated using the formula: STTGMA_LE_ = 1/ (1 + EXP(-(−3.4135 + 0.0450*Age + 0.2494*CCI – 0.3278*GCS + 0.5104*AIS_Head_and_Neck + 0.4163*AIS_Chest))). Age and Charlson Comorbidity Index were measured as continuous variables. GCS score, scored from 3 to 15, was categorized as mild (13–15), moderate (8–12), and severe (3–7), and also measured as a continuous variable. AIS, typically categorized as no injury, minor, moderate, serious, severe, critical, or maximal injuries, was scored from 0 to 6, respectively. The STTGMA score is computed as a logarithm of these four measures and ranges from 0 to 1. We reported the score as a percentage, with higher values indicating greater injury severity. We generated STTGMA risk categories – minimal (0–50%), low (51–80%), moderate (81–95%), and high (greater than 95%), using the percentile distribution of scores.

The secondary predictor variables were the GTOS and RTS. The GTOS was calculated using the formula: GTOS = Age + (2.5*ISS + 22 (if transfused with packed red blood cells within 24 hours. Age was measured as a continuous variable, and transfusion within 24 hours was measured as a binary variable – yes (score of 1) or no (score of 0). The ISS is scored on a scale of 0–75. It accounts for the severity of injuries to six body regions: the head and neck, face, chest, abdomen, extremities, and external regions (such as injuries to the skin or other external parts of the body that do not fall into the other five specific regions). Each of these body regions is assigned a score between 0 and 6, with 0, 1, 2, 3, 4, 5, and 6 representing no injury, minor, moderate, serious, severe/life-threatening, critical, and unsurvivable injuries, respectively. The top three scores across the six body regions are then squared and summed to calculate the final ISS. Any score of 6 in any body region upgrades the ISS to 75. The eventual GTOS was measured as a continuous variable, with higher scores indicating greater injury severity.

The RTS was calculated using the formula: RTS = (0.9368 * GCS category) + (0.7326 * Systolic Blood Pressure (SBP) category) + (0.2908 * Respiratory Rate (RR) category). Each of the three variables in the RTS was measured on a scale of 0–4. For the GCS category, points 0, 1, 2, 3, and 4 correspond to GCS scores of 3, 4–5, 6–8, 9–12, and 13–15, respectively. For the SBP category, points 0, 1, 2, 3, and 4 correspond to SBP values of 0, 1–49, 50–75, 76–89, and more than 89, respectively. For the RR category, points 0, 1, 2, 3, and 4 correspond to RR of 0, 1–5, 6–9, more than 29, and 10–29, respectively. The RTS was measured as a continuous variable, with lower scores indicating higher injury severity.

### Control variables

We did not control for any additional variables in the model prediction and ROC analysis. For the secondary analysis, we controlled for sex, race/ethnicity, body mass index, insurance type, and a history of repeated fall injury. Sex was defined as male or female. Race/ethnicity was defined as non-Hispanic White, non-Hispanic Black, Hispanic, Asian, and other races. Body mass index was defined as normal weight (18.0–24.9 kg/m^2^), underweight (<18.0 kg/m^2^), overweight (25.0–29.9 kg/m^2^), and obese (≥30.0 kg/m^2^). Insurance type was defined as a three-level categorical variable: Medicare/Medicaid, other health insurance, and no health insurance. A history of repeated fall injury was measured as a binary variable – yes or no.

### Data analysis

We reported the frequency distribution and summary statistics in the entire, training, and test datasets. We reported differences between the training and test datasets using the chi-square test, independent-samples t-test, and Mann-Whitney U test, as appropriate. We also reported the frequency distributions and summary statistics by STTGMA risk categories. We computed median differences in hospital length of stay and assessed standardized differences across predictor and control variables using Cohen’s d [40]. Also, we computed case-fatality rates for the predictor and control variables and assessed differences between them using the log-rank test.

For model prediction and ROC analysis, we first assessed the logarithmic relationship between in-hospital death and the three predictor variables—STTGMA risk-triage scores, GTOS, and RTS. Next, we generated the predicted estimates for each of the three regression models. Thereafter, we calculated the area under the ROC (AUROC) using sensitivity on the y-axis and 1-specificity on the x-axis, and reported the accuracy and 95% confidence interval (CI). AUROC values between 0.7 and 0.8 are interpreted as acceptable, 0.8 to 0.9 as excellent, and higher than 0.9 as outstanding [41]. We compared the AUROC values of the three risk triage tools using the DeLong test and reported the associated p-values [42]. P-values less than 0.05 indicate statistically significant differences in the predictive accuracies of the risk triage tools. Also, we computed the Youden index – a measure of a tool’s effectiveness as a diagnostic marker [32]. The Youden index ranges from 0 to 100% and values of 50% or higher are considered adequate [43].

For the secondary analysis, we performed quantile regression analyses to assess the association between the STTGMA risk categories and hospital length of stay. We reported the unadjusted and adjusted median differences and the 95% CI. We also performed a time-varying Cox proportional hazard regression analysis to assess the differences between the STTGMA risk categories and the time-to-death from fall-related injuries. We selected a time-varying model since the test of proportionality of strata was significant [44]. We reported the unadjusted and adjusted hazard risk ratios (mortality risk ratios) and their 95% CIs.

### Human subject research

We obtained Institutional Review Board (IRB) approval from the New York University Langone Health IRB (i20_01316_MOD05). Since this study is a secondary data analysis of health records, no informed consent was obtained from patients whose data were pooled. The authors did not have access to information that could identify individual participants during or after data collection.

## Results

### Descriptive characteristics

Among the 6,458 older adults who sustained fall-related injuries, the mean (SD) age was 80.7 (8.9) years (Table 1). The population was predominantly female (66%) and non-Hispanic White (61%). Most patients were either normal weight (40%) or overweight (36%). The injury mechanism was low energy (82%), and 97% of the population had mild GCS scores. Overall, 30% had head and neck injuries, 17% had facial injuries, 13% had chest injuries, 3% had abdominal injuries, 60% had injuries to the extremities, and 49% had injuries to external regions of the body. Also, 12% had a previous history of fall-related injuries, 42% of the population had no associated comorbidities, and 8% had blood transfusions within 24 hours of index admission. The median (Q1, Q3) hospital length of stay was 2 (0.0, 5.0) days, while the median (Q1, Q3) STTGMA risk score, GTOS, and RTS scores were 1.6% (0.9%, 3.1%), 93.0 (83.1, 106.0), and 7.8 (7.8, 7.8), respectively. There were no significant differences in demographic, injury, or hospital disposition characteristics between the training and test datasets.

**Table 1:** Demographic and injury characteristics of the study population in the training and test datasets among older adult trauma patients involved in fall-related injuries (N = 6,458).

Variables	Total population (N = 6,458)	Training set (n = 4,521)	Test set (n = 1,937)	p-value*
Age in years				
(Mean (SD)) ^†, ‡^	80.7 (8.9)	80.8 (8.9)	80.6 (8.9)	0.518
Sex^§^				
Female	4249 (65.8)	3001 (66.4)	1248 (64.4)	0.130
Male	2209 (34.2)	1520 (33.6)	689 (35.6)	
Race/Ethnicity^§^				
Non-Hispanic White	3957 (61.3)	2769 (61.2)	1188 (61.3)	0.872
Non-Hispanic Black	444 (6.9)	307 (6.8)	137 (7.1)	
Non-Hispanic Asian	119 (1.8)	79 (1.8)	40 (2.1)	
Hispanic	971 (15.0)	688 (15.2)	283 (14.6)	
Other Races	967 (15.0)	678 (15.0)	289 (14.9)	
Body Mass Index Categories^§^				
Normal Weight	2582 (40.0)	1808 (40.0)	774 (39.9)	0.292
Underweight	387 (6.0)	258 (5.7)	129 (6.7)	
Overweight	2293 (35.5)	1629 (36.0)	664 (34.3)	
Obese	1196 (18.5)	826 (18.3)	370 (19.1)	
Insurance Type^§^				
Medicare/Medicaid	3,627 (56.2)	2,530 (56.6)	1,097 (56.6)	0.619
Other insurance types	2,669 (41.3)	1,881 (41.6)	788 (40.7)	
No insurance	162 (2.5)	53 (2.7)	53 (2.7)	
Injury type^§,‡^				
Low energy	5313 (82.3)	3731 (82.5)	1582 (81.7)	0.411
High energy	1145 (17.7)	790 (17.5)	355 (18.3)	
Glasgow Coma Scale Score^§,‡^				
Mild	6260 (96.9)	4383 (96.9)	1877 (96.9)	0.992
Moderate	123 (1.9)	86 (1.9)	37 (1.9)	
Severe	75 (1.2)	52 (1.2)	23 (1.2)	
Head and Neck Injury				
No	4549 (70.4)	3210 (71.0)	1339 (69.1)	0.130
Yes	1909 (29.6)	1311 (29.0)	598 (30.9)	
Injury to the Face				
No	5372 (83.2)	3759 (83.2)	1613 (83.3)	0.900
Yes	1086 (16.8)	762 (16.8)	324 (16.7)	
Chest Injury				
No	5638 (87.3)	3940 (87.2)	1698 (87.7)	0.571
Yes	820 (12.7)	581 (12.8)	239 (12.3)	
Injury to the Abdomen				
No	6242 (96.7)	4373 (96.7)	1869 (96.5)	0.627
Yes	216 (3.3)	148 (3.3)	68 (3.5)	
Injury to Extremities				
No	2574 (39.9)	1770 (39.1)	804 (41.5)	0.076
Yes	3884 (60.1)	2751 (60.9)	1133 (58.5)	
External Cause of Injury				
No	3285 (50.9)	2312 (51.1)	973 (50.2)	0.504
Yes	3173 (49.1)	2209 (48.9)	964 (49.8)	
History of Previous Fall Injury				
No	5655 (87.6)	3960 (87.6)	1695 (87.5)	0.925
Yes	803 (12.4)	561 (12.4)	242 (12.5)	
Charlson Comorbidity Index^§,‡^				
No comorbidity	2695 (41.7)	1903 (42.1)	792 (40.9)	0.466
1 comorbidity	2257 (35.0)	1579 (34.9)	678 (35.0)	
2 comorbidities	1002 (15.5)	701 (15.5)	301 (15.5)	
3 or more comorbidities	504 (7.8)	338 (7.5)	166 (8.6)	
Transfusion within 24 hours				
No	5929 (91.8)	4157 (91.9)	1772 (91.5)	0.531
Yes	529 (8.2)	364 (8.1)	165 (8.5)	
In-hospital Mortality^§^				
Alive	6328 (98.0)	4433 (98.1)	1897 (97.9)	0.698
Dead	130 (2.0)	89 (1.9)	41 (2.1)	
Hospital Length of Stay^¶^				
Median Days (Q1, Q3)	2.0 (0.0, 5.0)	2.0 (0.0, 5.0)	2.0 (0.0, 5.0)	0.890
Mean Days (SD)	3.4 (6.1)	3.4 (6.6)	3.3 (4.8)	0.667
STTGMA Risk Score (%)^¶^				
Mean Score (SD)	3.4 (7.1)	3.4 (6.9)	3.4 (7.4)	0.758
Median Score (Q1, Q3)	1.6 (0.9, 3.1)	1.6 (0.9, 3.1)	1.6 (0.9, 3.2)	0.751
STTGMA categories^§^				
Minimal Risk	3229 (50.0)	2262 (50.0)	967 (49.9)	0.714
Low Risk	1937 (30.0)	1367 (30.2)	570 (29.4)	
Moderate Risk	969 (15.0)	664 (14.7)	305 (15.8)	
High Risk	323 (5.0)	228 (5.0)	95 (4.9)	
Geriatric Trauma Outcome Score				
Mean (SD)	96.7 (18.8)	96.5 (18.5)	97.0 (19.3)	0.322
Median (Q1, Q3)	93.0 (83.1, 106.0)	93.1 (83.1, 105.8)	92.6 (83.0, 106.5)	0.764
Revised Trauma Score				
Mean (SD)	7.8 (0.4)	7.8 (0.3)	7.8 (0.4)	0.179
Median (Q1, Q3)	7.8 (7.8, 7.8)	7.8 (7.8, 7.8)	7.8 (7.8, 7.8)	0.234

† Independent sample t-test performed; ‡ Variables in the STTGMA calculation; § Chi-square test performed; ¶ Mann-Whitney U test performed; AIS: Abbreviated Injury Scale; SD: Standard Deviation; Normal weight: 18.5–24.9 kg/m^2^; Underweight: < 18.5 kg/m^2^; Overweight: 25.0–29.9 kg/m^2^, Obese: ≥ 30.0 kg/m^2^; Glasgow Coma Scale score: Categorized as mild (13–15), moderate (8–12), and severe (3–7); High-energy falls: Falls from heights greater than two meters or collisions with an automobile; Low-energy falls: Falls from a standing height or lower. *The p-value was calculated to compare characteristics between the training and test datasets; the absence of a statistically significant difference indicates that the simple random sampling produced balanced groups.

**Fig 2:**
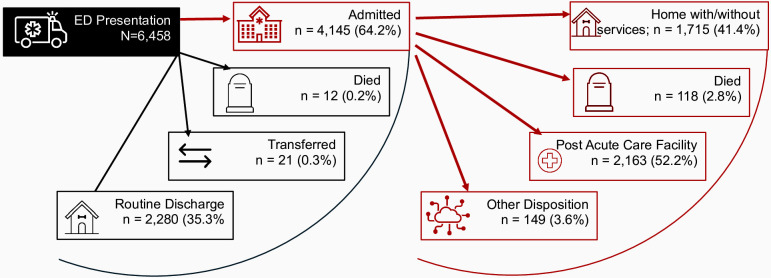
Clinical care trajectory of older adults with fall-related injuries. Home with/without services include routine discharge to home/self-care, home with services. Died includes death after withdrawal of care, as full code, as unknown, as care not begun DNR/DNI, or as met brain death criteria. Post Acute Care Facility includes inpatient rehabilitation, sub-acute inpatient rehabilitation, traumatic brain rehabilitation, spinal cord injury rehabilitation, new or return to skilled nursing facility, and hospice. Other disposition includes left against medical advice, hospice, homeless shelter.

The clinical trajectory of the 6,458 older adults that presented to the ED with fall-related injuries is shown in Fig 2 (Fig 2). Of these, 4,145 (64.2%) were admitted to the hospital, 2,280 (35.3%) were discharged home from the ED, 21 (0.3%) were transferred to another facility, and 12 (0.2%) died in the ED. Among admitted patients, 1,715 (41.4%) were discharged home with or without home health services, 2,163 (52.2%) were discharged to a post-acute care facility, and 118 (2.8%) died during hospitalization. Hence, a total of 130 (2.0%) older adults died during their fall-related hospital visit.

We assessed differences in sociodemographic, clinical, and injury characteristics by STTGMA risk categories – minimal, low, moderate, and high (Table 2). The proportion of male patients increased from 34% in the minimal risk group to 43% in the high-risk group. The proportion of Hispanic patients also increased from 1.9% in the minimal risk to 2.5% in the high-risk group. Also, the proportion of older adults who were underweight increased from 4.2% in the minimal risk group to 9% in the high-risk group. Approximately 3% of older adults without insurance were in the minimal risk group, while 5% of older adults without insurance were in the high-risk group. Furthermore, 10% of the minimal risk group had previous falls, while 13% of those in the high-risk group had previous falls. The median GTOS scores increased stepwise across the STTGMA risk categories, and the proportion of in-hospital mortality increased from 0.5% in the minimal risk group to 16% in the high-risk group. The median hospital length of stay increased from 1 day in the minimal risk group to 4 days in the high-risk group.

**Table 2:** Demographic and injury characteristics of older adult trauma patients involved in fall-related injuries by STTGMA risk categories (N = 6,458).

Variables	STTGMA risk categories			
	Minimal risk n = 3,229 (%)	Low risk n = 1,937 (%)	Moderate risk n = 969 (%)	High risk n = 323 (%)
Sex				
Female	2,120 (65.7)	1,301 (67.2)	643 (66.4)	185 (57.3)
Male	1,109 (34.3)	636 (32.8)	326 (33.6)	138 (42.7)
Race/Ethnicity				
Non-Hispanic Whites	1,936 (60.0)	1,220 (63.0)	617 (63.7)	184 (57.0)
Non-Hispanic Blacks	248 (7.7)	129 (6.7)	53 (5.5)	14 (4.3)
Non-Hispanic Asians	538 (16.7)	274 (14.1)	104 (10.7)	55 (17.0)
Hispanic	62 (1.9)	30 (1.5)	19 (2.0)	8 (2.5)
Other Races	445 (13.8)	284 (14.7)	176 (18.1)	62 (19.2)
Body Mass Index Categories				
Normal Weight	1,093 (33.8)	865 (44.7)	460 (47.4)	164 (50.8)
Underweight	135 (4.2)	138 (7.1)	85 (8.8)	29 (9.0)
Overweight	1,285 (39.8)	619 (32.0)	304 (31.4)	85 (26.3)
Obese	716 (22.2)	315 (16.3)	120 (12.4)	45 (13.9)
Insurance Type				
Medicare/Medicaid	1,672 (51.8)	1,179 (60.9)	581 (60.0)	195 (60.4)
Other insurance types	1,471 (45.5)	723 (37.3)	364 (37.5)	111 (34.4)
No insurance	86 (2.7)	35 (1.8)	24 (2.5)	17 (5.2)
History of Previous Fall Injury				
No	2,899 (89.8)	1,656 (85.5)	820 (84.6)	280 (86.7)
Yes	330 (10.2)	281 (14.5)	149 (15.4)	43 (13.3)
Geriatric Trauma Outcome Score				
Median (Q1, Q3)	84.8 (78.3, 94.1)	96.1 (89.2, 106.9)	107.3 (99.1, 118.5)	124.3 (109.0, 142.7)
Revised Trauma Score				
Median (Q1, Q3)	7.84 (7.84, 7.84)	7.84 (7.84, 7.84)	7.84 (7.84, 7.84)	7.84 (6.90, 7.84)
In-hospital Mortality^§^				
Alive	3,212 (99.5)	1,908 (98.5)	939 (96.9)	271 (83.9)
Dead	17 (0.5)	29 (1.5)	30 (3.1)	52 (16.1)
Hospital Length of Stay^¶^				
Median Days (Q1, Q3)	1.0 (0.0, 4.0)	3.0 (0.0, 5.0)	4.0 (2.0, 6.0)	4.0 (2.0, 8.0)

Higher STTGMA risk categories were associated with progressively longer hospital stays, with small standardized differences ranging from −0.10 to −0.35 compared to the minimal risk group (Table 3). Also, there were significant differences in the fall case fatality rates by sex (p < 0.001), insurance type (p = 0.033), history of previous fall-related injuries (p = 0.035), and STTGMA risk categories (p < 0.001). Those classified as having minimal injury risk had a fall case fatality rate of 0.5%. The case fatality rates increased stepwise to 1.5%, 3.1%, and 16.1% for those classified as low risk, moderate risk, and high risk, respectively.

**Table 3:** Summary statistics of the length of stay and incidence rates of fatal fall-related injuries across the study population (N = 6,458).

Variables	Length of stay		Mortality	
	Median (days) (Q1, Q3)	Standardized effect size^‡^	Case fatality rate (%) (95% CI)	p-value^§^
Overall	2.0 (0.0, 5.0)		1.98 (1.67, 2.35)	
Sex*				
Female	2.0 (0.0, 5.0)	Ref	1.27 (0.97, 1.66)	<0.001*
Male	2.0 (0.0, 5.0)	-0.07	3.35 (2.68, 4.19)	
Race/Ethnicity				
Non-Hispanic Whites	2.0 (0.0, 5.0)	Ref	2.25 (1.83, 2.76)	0.081
Non-Hispanic Blacks	2.0 (0.0, 4.0)	0.12	0.45 (0.11, 1.78)	
Non-Hispanic Asians	2.0 (0.0, 4.0)	0.04	2.52 (0.82, 7.53)	
Hispanic	1.0 (0.0, 4.0)	0.16	1.13 (0.63, 2.03)	
Other Races	2.0 (0.0, 5.0)	0.08	2.38 (1.59, 3.55)	
Body Mass Index Categories				
Normal Weight	3.0 (0.0, 5.0)	Ref	1.74 (1.30, 2.33)	0.522
Underweight	3.0 (0.0, 6.0)	-0.11	2.58 (1.40, 4.74)	
Overweight	2.0 (0.0, 4.0)	0.01	1.96 (1.47, 2.62)	
Obese	2.0 (0.0, 4.0)	-0.04	2.34 (1.62, 3.37)	
Insurance Type*				
Medicare/Medicaid	2.0 (0.0, 5.0)	Ref	2.29 (1.85, 2.83)	0.033*
Other insurance types	2.0 (0.0, 5.0)	-0.09	1.42 (1.04, 1.95)	
No insurance	1.0 (0.0, 3.0)	0.08	4.32 (2.07, 8.79)	
History of Previous Fall Injury*				
No	2.0 (0.0, 5.0)	Ref	2.14 (1.79, 2.55)	0.035*
Yes	2.0 (0.0, 5.0)	0.01	0.87 (0.42, 1.82)	
STTGMA categories*				
Minimal Risk	1.0 (0.0, 4.0)	Ref	0.53 (0.33, 0.85)	<0.001*
Low Risk	3.0 (0.0, 5.0)	-0.10	1.50 (1.04, 2.15)	
Moderate Risk	4.0 (2.0, 6.0)	-0.26	3.10 (2.17, 4.39)	
High Risk	4.0 (2.0, 8.0)	-0.35	16.10 (12.48, 20.62)	

§: Log-rank test performed; ‡: Effect size, Cohen’s D, computed by assessing the bivariate standardized difference between the predictor variables and the log-transformed length of stay. Effect size: < 0.01 = minimal, 0.0–0.19 = negligible, 0.20–0.49 = small effect size, 0.50–0.79 = moderate effect size, ≥ 0.80 = large effect size *: Significant values suggest that there is a lack of proportionality across the strata (failed test of proportionality); STTGMA: Score for Trauma Triage in the Geriatric and Middle-Aged.

### Model prediction and ROC analysis

Using the training dataset, a unit increase in STTGMA risk score was associated with a 7% increased odds of fatal fall injury (OR: 1.07; 95% CI: 1.06–1.08) and this estimate remained unchanged after adjusting for sex, race/ethnicity, body mass index, insurance type, and history of previous fall injury (Table 3). The model predicted 16.5% of the variability in death in the population in the training data set. A unit increase in the GTOS was associated with a 5% adjusted odds of fatal fall injury (Adjusted OR: 1.05; 95% CI: 1.04–1.06), and the GTOS model predicted 17% of the variability in death in the population in the training data set. A unit increase in the RTS was associated with a 78% reduced adjusted odds of fatal fall injury (Adjusted OR: 0.22; 95% CI: 0.17–0.29), and the RTS model predicted 22% of the variability in death in the population in the training data set.

**Fig 3:**
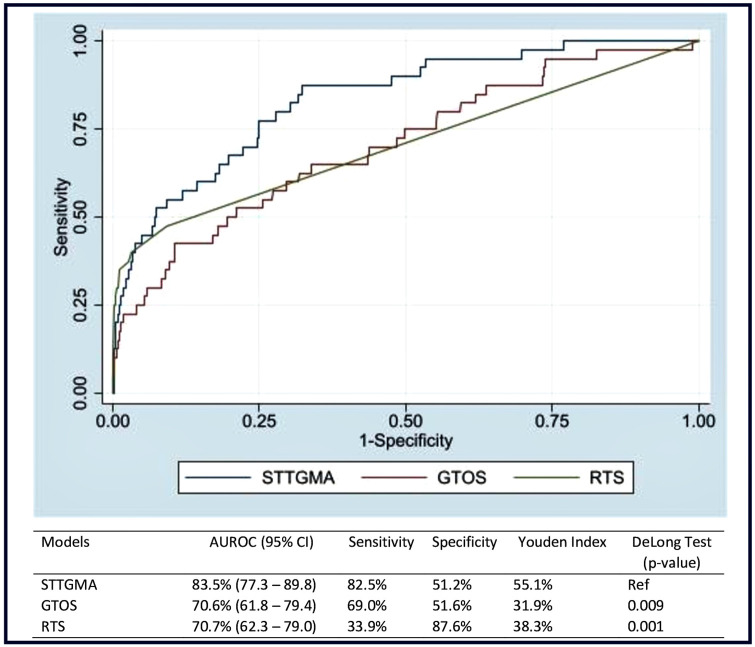
Diagnostic accuracy, sensitivity, and specificity of the Score for Trauma Triage in Geriatric and Middle Age (STTGMA), Geriatric Trauma Outcome Score (GTOS), and Revised Trauma Score (RTS) in predicting fall-related mortality among older adults with fall-related injuries. Youden Index: Ranges from 0 to 100% assuming sensitivity and specificity are of equal value; the higher the value, the better the discriminant property, and values of 50% or higher are acceptable; AUROC ranging from 0.7 to 0.8 is acceptable, 0.8 to 0.9 is excellent, and values higher than 0.9 as outstanding; AUROC: Area Under Receiver Operating Characteristic. For the DeLong test, the STTGMA was compared separately with GTOS and RTS. Significant differences in the AUROC between STTGMA and GTOS and STTGMA and RTS were reported using the p-values, P-values less than 0.05 suggest that the differences are statistically significant.

Using the test data, the STTGMA risk score demonstrated 84% accuracy in predicting in-hospital fall-related mortality (95% CI: 77.3–89.8) (Fig 3). The sensitivity and specificity of the STTGMA risk score were 83% and 51%, respectively, and the Youden index was 55%. The GTOS demonstrated 71% accuracy in predicting in-hospital fall-related mortality (95% CI: 61.8–79.4). The sensitivity and specificity of the GTOS were 69% and 52%, respectively, and the Youden index was 32%. Also, the RTS demonstrated 71% accuracy in predicting in-hospital fall-related mortality (95% CI: 62.3–79.0). The sensitivity and specificity of the RTS were 34% and 88%, respectively, and the Youden index was 38%. The diagnostic accuracy of STTGMA in predicting fall-related mortality was significantly higher than GTOS (p = 0.009) and RTS (p = 0.001).

### Hospital length of stay

We report the unadjusted median change in the hospital length of stay across the demographic characteristics and STTGMA risk categories (Table 4). In the adjusted models, non-Hispanic Blacks (Adjusted Median Difference (aMD): −1.0; 95% CI: −1.6 – −0.4) and Hispanics (aMD: −1.0; 95% CI: −1.4 – −0.6) had significantly shorter hospital length of stays compared to those who were non-Hispanic White. Compared to the minimal risk category, patients classified as low risk had a two-day median increase in their hospital length of stay (95% CI: 1.7–2.3). Patients classified as moderate (95% CI: 2.6–3.4) and high-risk (95% CI: 2.4–3.6) categories had a three-day median increase in their hospital length of stay.

**Table 4:** Summary of the unadjusted and adjusted odds ratio assessing the relationship with death using the training data (N = 4,521).

Models*	Unadjusted odds ratio (95% CI)	Adjusted odds ratio (95% CI)	Adjusted R2 (%)
STTGMA	1.07 (1.06–1.08)	1.07 (1.06–1.09)	16.50%
GTOS	1.04 (1.04–1.05)	1.05 (1.04–1.06)	17.15%
RTS	0.23 (0.19–0.30)	0.22 (0.17–0.29)	21.74%

*Models: Each of the predictor variables represents one model each with the outcome being death. Each model adjusted for sex, race/ethnicity, body mass index, insurance type, and history of previous fall injury; STTGMA: Score for Trauma Triage in Geriatric and Middle Age; GTOS: Geriatric Trauma Outcome Score; RTS: Revised Trauma Score

### Time to death

We report the unadjusted mortality risk ratio (hazard risk) across demographic characteristics and STTGMA risk categories (Table 5). In the adjusted models, males had two times the mortality risk compared to females (adjusted Hazard Ratio (aHR): 1.97; 95% CI: 1.33–2.93). Those who are obese had 1.9 times the mortality risk as compared to those who are of normal weight (aHR: 1.92; 95% CI: 1.14–3.23). Compared to those on Medicaid or Medicare, those with other insurance types had 47% lower adjusted mortality risk (aHR: 0.53; 95% CI: 0.29–0.97) while those without insurance had 20 times the adjusted mortality risk (aHR: 20.20; 95% CI: 1.94–210.14). Also, those classified as having low risk had 3.1 times (95% CI: 1.29–7.39) the mortality risk compared to those classified as minimal risk, and the mortality risk ratio increased to 3.8 (95% CI: 1.55–9.15) and 29.5 (95% CI: 12.30–70.78) in the moderate and high-risk categories.

**Table 5:** Summary of the unadjusted and adjusted quantile regression and time-varying Cox proportional hazard regression assessing the changes in length of stay and time-to-death among the study population (N = 6,458).

Variables	Length of stay		Mortality risk	
	Unadjusted coefficient (95% CI)	Adjusted coefficient (95% CI)	Unadjusted hazard ratio (95% CI)	Adjusted hazard ratio^##^ (95% CI)
STTGMA categories				
Minimal Risk	Ref	Ref	Ref^#^	Ref^#^
Low Risk	2.0 (1.73, 2.27)	2.0 (1.66, 2.34)	2.89 (1.23, 6.77)	3.09 (1.29, 7.42)
Moderate Risk	3.0 (2.66, 3.34)	3.0 (2.57, 3.43)	3.77 (1.59, 8.94)	3.94 (1.63, 9.54)
High Risk	3.0 (2.45, 3.54)	3.0 (2.32, 3.68)	25.93 (11.42, 58.90)	30.20 (12.60, 72.35)
Sex*				
Female	Ref	Ref	Ref	Ref^#^
Male	2.0 (1.76, 2.24)	0.0 (−0.31, 0.31)	2.00 (1.37, 2.91)	1.89 (1.28, 2.79)
Race/Ethnicity*				
Non-Hispanic Whites	Ref	Ref	Ref^#^	Ref
Non-Hispanic Blacks	0.0 (−0.55, 0.55)	−1.0 (−1.59, −0.41)	0.03 (0.00, 1.51)	0.03 (0.00, 1.45)
Non-Hispanic Asians	−1.0 (−1.39, −0.61)	−1.0 (−1.43, −0.57)	0.70 (0.28, 1.72)	0.48 (0.19, 1.24)
Hispanic	0.0 (−1.02, 1.02)	−1.0 (−2.09, 0.09)	0.22 (0.02, 3.11)	0.06 (0.00, 1.15)
Other Races	0.0 (−0.39, 0.39)	0.0 (−0.43, 0.43)	1.24 (0.63, 2.46)	0.96 (0.47, 1.95)
Body Mass Index Categories*				
Normal Weight	Ref	Ref	Ref	Ref
Underweight	0.0 (−0.51, 0.51)	0.0 (−0.64, 0.64)	1.22 (0.59, 2.51)	1.67 (0.79, 3.53)
Overweight	−1.0 (−1.27, −0.73)	0.0 (−0.34, 0.34)	1.28 (0.83, 1.97)	1.52 (0.97, 2.37)
Obese	−1.0 (−1.33. −0.67)	0.0 (−0.41. 0.41)	1.39 (0.84, 2.29)	1.86 (1.11, 3.11)
Insurance Type				
Medicare/Medicaid	Ref	Ref	Ref^#^	Ref^#^
Other insurance types	0.0 (−0.34, 0.34)	0.0 (−0.31, 0.31)	0.46 (0.26, 0.81)	0.52 (0.29, 0.95)
No insurance	−1.0 (−2.07, 0.07)	0.0 (−0.94, 0.94)	41.18 (3.80, 446.46)	20.51 (1.98, 212.62)
History of Previous Fall Injury				
No	Ref	Ref	Ref	Ref^#^
Yes	0.0 (−0.35, 0.35)	0.0 (−0.44, 0.44)	0.44 (0.21, 0.95)	0.43 (0.20, 0.94)

STTGMA: Score for Trauma Triage in the Geriatric and Middle-Aged; #Time varying Cox proportional hazard model performed: Interaction of time X male, time X insurance type, time X history of previous fall injury, and time X STTGMA risk categories created in the time-varying Cox proportional multivariable regression model; Normal weight: 18.5–24.9 kg/m^2^; Underweight: < 18.5 kg/m^2^; Overweight: 25.0–29.9 kg/m^2^, Obese: ≥ 30.0 kg/m^2^

## Discussion

This study provides a validated fall injury triage tool that can accurately predict fall-related mortality risk. We report that among adults 65 years and older with fall-related injuries, the STTGMA demonstrated high accuracy in predicting fatal fall-related mortality. Additionally, STTGMA demonstrated superior diagnostic accuracy compared to the GTOS and the RTS in predicting fatal fall-related mortality. Using data from a single-level I trauma center, the fall case fatality rate and time-to-death exponentially increased in a dose-response pattern across the STTGMA-derived risk categories. Also, STTGMA risk classifies the hospital length of stay into three non-overlapping categories – minimal risk, low risk, and moderate/high-risk.

The STTGMA risk triage tool demonstrated excellent accuracy in predicting fatal fall-related mortality. Earlier studies have reported the high discriminative ability of the STTGMA in predicting in-hospital mortality among older adults with hip fractures and among patients who had orthopedic and neurosurgical consultations [25,26]. Since hip fractures represent 10–15 percent of the complications that occur following fall-related injuries [45], our study extended the utility of STTGMA for the larger older adult trauma population with fall-related injuries.

The notable discrepancy in diagnostic accuracy observed when comparing the STTGMA with GTOS and RTS may be attributed to the selection of covariates within their respective designs and scoring methodologies. Haider et al. [46] identified five pivotal survival covariates essential for trauma research: age, sex, anatomical severity, physiological severity, and injury mechanism. The GTOS incorporates only age and anatomical severity (injury severity score) [11,15], whereas the RTS considers only physiological severity (systolic blood pressure, respiratory rate, and Glasgow Coma Scale) [12,16,47,48]. In contrast, the STTGMA risk triage tool incorporates four of the five survival covariates (age, anatomical severity, physiological severity, and injury mechanism) in its scoring algorithm. Another geriatric-focused risk assessment tool is the Elderly Mortality After Trauma (EMAT) score [19]. The EMAT score, available in both quick (qEMAT) and full (fEMAT) versions, is computed using eight and 26 variables, respectively. It captures four of the five key survival covariates, excluding sex. We were unable to compare the STTGMA with the EMAT scores because the required EMAT variables were unavailable in our institutional trauma databases.

While the exponential increase in mortality risk from minimal to low, moderate, and high-risk categories lends credence to the STTGMA triage tool’s predictive ability, it also provides the opportunity to identify the at-risk population and create risk-specific interventions for these patients. Identifying community clusters of high-risk patient populations for fall-related injuries and implementing evidence-based interventions, such as the Stopping Elderly Accidents, Deaths & Injuries (STEADI) [49–51] may reduce county- and state-level fall-related mortality rates. Additionally, the Emergency Medical Service may embed the STTGMA risk scoring into their injury triage assessment and institute policies to ensure rapid transfer of older adults at risk of fatal fall-related injuries to levels I and II trauma centers.

The STTGMA risk triage tool also offers clinical benefits. By accurately identifying older adults at the highest risk of fatal fall-related injuries, the STTGMA risk triage tool can be used to guide decisions on trauma team activation. The early and accurate activation of the trauma team will reduce geriatric trauma under-triage rates. It may improve the trauma outcomes of older adults with fall and non-fall-related injuries [8,52–55]. Also, the STTGMA risk stratification tool can help determine the appropriate admission location, whether it is the general hospital floor, a high-dependency unit, or the intensive care unit. Following in-hospital admission, the mortality risk stratification can assist in making informed pre-operative decisions, facilitate difficult discussions with patients, families, and caregivers, and inform the need for multidisciplinary care. The emergency, trauma, and geriatric departments can use the STTGMA risk stratification and mortality prediction to motivate the prevention of such adverse events. Additionally, departmental leaders can leverage information on fall-related mortality risk prediction to enhance in-hospital and community-level awareness of fall prevention, provide educational training for healthcare professionals, revise departmental protocols and guidelines as needed, and actively oversee the department’s and institution’s performance in the assessment and outcomes of geriatric trauma cases. Lastly, the STTGMA risk triage tool can be incorporated into risk modeling, further advancing the knowledge of clinical processes that can improve injury survival among older adult trauma patients.

This study has its limitations. Data entry errors, unreported history of falls, and inaccurately recorded history of background chronic medical conditions may influence the results of this study. Also, our study is from a single institutional trauma database, and our results may not be generalizable to other trauma centers. Additionally, our findings may not be generalizable to older adults treated in non-academic or rural trauma settings, who may differ in their baseline health status, prehospital time, or injury management protocols [56,57]. The STTGMA risk triage scoring is computationally complex, and this may deter physicians and other providers from using this powerful predictive tool. Healthcare institutions may subvert this challenge by integrating the publicly available tool into their electronic health record system and automating the risk calculations for trauma patients 65 years and older. Since the risk categorization is defined using percentiles, every healthcare system that wishes to integrate the STTGMA into its departmental workflow will have to use its retrospective data as a primer to generate the STTGMA risk categories. However, the ability to adapt the STTGMA risk scoring to different patient populations is one of the unique qualities of the risk triage tool. This study addresses the need for a highly predictive risk triage tool for older adult trauma patients, and it is the first to report the predictive accuracy of the STTGMA risk triage tool among older adults with fall-related injuries.

## Conclusion

STTGMA predicts in-hospital mortality from fall-related injuries with high accuracy and can risk-stratify hospital length of stay and time to death in older adults. Its integration into predictive tools like PersonaCARE [58], which automates data extraction and triage decision-making for qualifying patients presenting in the ED, demonstrates its feasibility for real-time clinical use and supports value-based care. By identifying older adults at high risk of fatal outcomes, STTGMA can guide in-hospital care protocols and inform targeted interventions to improve patient outcomes and value-based care.

## Supporting information

S1 File STROBE checklist. (DOCX)

S2 File PLOS One clinical studies checklist. (DOCX)
