# Supplementary material for: Diagnostic accuracy and risk stratification of the score for trauma triage in the geriatric and middle-aged among older adults with fall-related injuries
Source: PLoS One. 2025 Dec 18;20(12):e0338948. doi: 10.1371/journal.pone.0338948 (PMC12714260; doi:10.1371/journal.pone.0338948)
Supplement: S1 File — (DOCX) [file pone.0338948.s001.docx]

STROBE Statement—checklist of items that should be included in reports of observational studies

|  | Item No. | Recommendation | Page  No. | Relevant text from manuscript |
| --- | --- | --- | --- | --- |
| **Title and abstract** | 1 | (*a*) Indicate the study’s design with a commonly used term in the title or the abstract | 2 | Using a retrospective cohort design, we selected 5,791 geriatric trauma patients… |
|  |  | (*b*) Provide in the abstract an informative and balanced summary of what was done and what was found | 2 | The outcome variable was fatal fall injury, measured as a binary variable. The predictor variable was the STTGMA score, measured as a continuous variable and a four-level categorical variable. We report the predictive accuracy (95% confidence interval (CI)) of the STTGMA. We further assessed the relationship between the STTGMA risk categories and hospital length of stay and time-to-death by performing multivariable quantile regression and time-varying Cox proportional hazard analyses, respectively. |
| Introduction | | | |  |
| Background/rationale | 2 | Explain the scientific background and rationale for the investigation being reported | 3 | Trauma-specific scales such as the Ohio geriatric guideline have demonstrated 52 to 65% accuracy in predicting level I and II trauma center transfer while the Manchester triage guideline showed a 74% accuracy in predicting geriatric trauma mortality.(13, 14). However, these trauma scales have not been used specifically for fall-related mortality. |
| Objectives | 3 | State specific objectives, including any prespecified hypotheses | 4 | This study aims, therefore, to assess the diagnostic accuracy of the STTGMA risk triage tool in predicting in-hospital mortality and to assess the association between the STTGMA-derived risk categories and hospital length of stay and time-to-death from fall-related injuries |
| Methods | | | |  |
| Study design | 4 | Present key elements of study design early in the paper | 4 | Using a retrospective cohort design, we pooled trauma registry data between 2017 and 2021 from a single Level-I trauma center that serves a racially diverse community in New York. |
| Setting | 5 | Describe the setting, locations, and relevant dates, including periods of recruitment, exposure, follow-up, and data collection | 4 | Using a retrospective cohort design, we pooled trauma registry data between 2017 and 2021 from a single Level-I trauma center that serves a racially diverse community in New York. |
| Participants | 6 | (*a*) *Cohort study*—Give the eligibility criteria, and the sources and methods of selection of participants. Describe methods of follow-up  *Case-control study*—Give the eligibility criteria, and the sources and methods of case ascertainment and control selection. Give the rationale for the choice of cases and controls  *Cross-sectional study*—Give the eligibility criteria, and the sources and methods of selection of participants | 4-5 | We selected adults, 55 years and older, who presented to the ED with traumatic injuries (N=7,634) (Figure 1). We included patients between the ages of 55 and 64 because trauma-related mortality significantly increases after age 55.(24) We excluded patients whose injuries were not related to falls (n=1,102). Duplicate entries (recurrent within-patient ED visits) were identified and we used the most recent encounter (n=740). We performed a listwise deletion for variables with missing entries when the proportion was less than 1% (variable: sex; n=1 (0.0%)). The final analysis included 5,791 adults aged 55 years and older who presented to the ED with fall-related injuries. |
|  |  | (*b*) *Cohort study*—For matched studies, give matching criteria and number of exposed and unexposed  *Case-control study*—For matched studies, give matching criteria and the number of controls per case |  |  |
| Variables | 7 | Clearly define all outcomes, exposures, predictors, potential confounders, and effect modifiers. Give diagnostic criteria, if applicable | 5-7 | Example text:  The primary outcome measure of interest was in-hospital death. We defined in-hospital death as death occurring either in the ED or during the index admission |
| Data sources/ measurement | 8* | For each variable of interest, give sources of data and details of methods of assessment (measurement). Describe comparability of assessment methods if there is more than one group | 5-7 | See number 7 above |
| Bias | 9 | Describe any efforts to address potential sources of bias | 5 | We performed multiple imputations after confirming that the missing pattern was missing at random using Little's test |
| Study size | 10 | Explain how the study size was arrived at | 4-5 | We selected adults, 55 years and older, who presented to the ED with traumatic injuries (N=7,634) (Figure 1). We included patients between the ages of 55 and 64 because trauma-related mortality significantly increases after age 55.(24) We excluded patients whose injuries were not related to falls (n=1,102). Duplicate entries (recurrent within-patient ED visits) were identified and we used the most recent encounter (n=740). We performed a listwise deletion for variables with missing entries when the proportion was less than 1% (variable: sex; n=1 (0.0%)). The final analysis included 5,791 adults aged 55 years and older who presented to the ED with fall-related injuries. |

Continued on next page

| Quantitative variables | 11 | Explain how quantitative variables were handled in the analyses. If applicable, describe which groupings were chosen and why | 5-7 | See response to number 7 above |
| --- | --- | --- | --- | --- |
| Statistical methods | 12 | (*a*) Describe all statistical methods, including those used to control for confounding | 7-8 | Example text:  For the secondary analysis, we performed a quantile regression analysis to assess the association between the STTGMA risk categories and hospital length of stay. We reported the unadjusted and adjusted median differences and the 95% CI. We also performed a time-varying Cox proportional hazard regression analysis to assess the differences between the STTGMA risk categories and the time-to-death from fall-related injuries. We selected a time-varying model since the test of proportionality of strata was significant, evidenced by the crossing of the log-log plot of the STTGMA risk categories, and the significant Schoenfeld residual proportional hazard test.(32) We reported the unadjusted and adjusted hazard risk ratio (mortality risk ratio) and the 95% CI. |
|  |  | (*b*) Describe any methods used to examine subgroups and interactions | 7-8 | See response to 11a above |
|  |  | (*c*) Explain how missing data were addressed | 5 | We performed multiple imputations after confirming that the missing pattern was missing at random using Little's test |
|  |  | (*d*) *Cohort study*—If applicable, explain how loss to follow-up was addressed  *Case-control study*—If applicable, explain how matching of cases and controls was addressed  *Cross-sectional study*—If applicable, describe analytical methods taking account of sampling strategy |  | NA |
|  |  | (*e*) Describe any sensitivity analyses |  | NA |
| Results | | | | |
| Participants | 13* | (a) Report numbers of individuals at each stage of study—eg numbers potentially eligible, examined for eligibility, confirmed eligible, included in the study, completing follow-up, and analysed | 21 | Figure 1 |
|  |  | (b) Give reasons for non-participation at each stage | 21 | Figure 1 |
|  |  | (c) Consider use of a flow diagram | 21 | Figure 1 |
| Descriptive data | 14* | (a) Give characteristics of study participants (eg demographic, clinical, social) and information on exposures and potential confounders | 8 | Example text:  Among the 5,791 adults who sustained fall injuries, the mean (SD) age was 77.5 (11.4) years (Table 1). The population was predominantly female (63%) and non-Hispanic White (63%). |
|  |  | (b) Indicate number of participants with missing data for each variable of interest | 5 | Three variables had missing values less than 10% - race/ethnicity (n=96, 1.7%), body mass index (n=295 (5.1%)), and Glasgow Coma Scale score (n=539, (9.3%)). |
|  |  | (c) *Cohort study*—Summarise follow-up time (eg, average and total amount) |  | NA |
| Outcome data | 15* | *Cohort study*—Report numbers of outcome events or summary measures over time | 8 | The median (Q1, Q3) STTGMA risk score was 1.3% (0.7%, 2.4%). The median (Q1, Q3) hospital length of stay was 2 (0.0, 5.0) days. There was a total of 122 (2.1%) in-hospital deaths. |
|  |  | *Case-control study—*Report numbers in each exposure category, or summary measures of exposure |  | NA |
|  |  | *Cross-sectional study—*Report numbers of outcome events or summary measures |  | NA |
| Main results | 16 | (*a*) Give unadjusted estimates and, if applicable, confounder-adjusted estimates and their precision (eg, 95% confidence interval). Make clear which confounders were adjusted for and why they were included | 9 | Example text:  We report the unadjusted median change in the hospital length of stay across the demographic characteristics and STTGMA risk categories (Table 3). In the adjusted models, non-Hispanic Black patients (Adjusted Median Difference (aMD): -1.0; 95% CI: -1.5 – -0.5) and Hispanic patients (aMD: -1.0; 95% CI: -1.3 – -0.7) had shorter hospital length of stays compared to those who were non-Hispanic White. |
|  |  | (*b*) Report category boundaries when continuous variables were categorized | 8 | We generated STTGMA risk categories – minimal (0 – 50%), low (51 – 80%), moderate (81 – 95%), and high (greater than 95%), using the percentile distribution of scores. |
|  |  | (*c*) If relevant, consider translating estimates of relative risk into absolute risk for a meaningful time period |  | NA |

Continued on next page

| Other analyses | 17 | Report other analyses done—eg analyses of subgroups and interactions, and sensitivity analyses | 8-9 | STTGMA risk score demonstrated 81% accuracy in predicting in-hospital mortality (95% CI: 75.7 - 86.5) (Figure 2A). The sensitivity and specificity of the STTGMA risk score were 80% and 51%, respectively, and the Youden index was 52%. |
| --- | --- | --- | --- | --- |
| Discussion | | | | |
| Key results | 18 | Summarise key results with reference to study objectives | 10 | STTGMA risk classifies the hospital length of stay into three non-overlapping categories of minimal risks, low to moderate risks, and high risks |
| Limitations | 19 | Discuss limitations of the study, taking into account sources of potential bias or imprecision. Discuss both direction and magnitude of any potential bias | 11 | Example text:  This study has its limitations. Data entry errors, unreported history of falls, and inaccurately recorded history of background chronic medical conditions may influence the results of this study. Additionally, our study is from a single institutional trauma database and result may not be generalizable to other trauma centers. |
| Interpretation | 20 | Give a cautious overall interpretation of results considering objectives, limitations, multiplicity of analyses, results from similar studies, and other relevant evidence | 12 | STTGMA predicts in-hospital mortality from fall injuries with high accuracy and can risk-stratify and predict hospital length of stay and time to death from fall injuries in older adults |
| Generalisability | 21 | Discuss the generalisability (external validity) of the study results | 11 | However, the ability to adapt the STTGMA risk scoring to different patient populations, is one of the unique qualities of the risk triage tool. This study addresses the need for a highly predictive risk triage tool for geriatric trauma patients and it is the first to report the predictive accuracy of the STTGMA risk triage tool among older adults with fall injuries |
| Other information | |  | | |
| Funding | 22 | Give the source of funding and the role of the funders for the present study and, if applicable, for the original study on which the present article is based |  | NA |

*Give information separately for cases and controls in case-control studies and, if applicable, for exposed and unexposed groups in cohort and cross-sectional studies.

**Note:** An Explanation and Elaboration article discusses each checklist item and gives methodological background and published examples of transparent reporting. The STROBE checklist is best used in conjunction with this article (freely available on the Web sites of PLoS Medicine at http://www.plosmedicine.org/, Annals of Internal Medicine at http://www.annals.org/, and Epidemiology at http://www.epidem.com/). Information on the STROBE Initiative is available at www.strobe-statement.org.
